# circRIP2 accelerates bladder cancer progression via miR-1305/Tgf-β2/smad3 pathway

**DOI:** 10.1186/s12943-019-1129-5

**Published:** 2020-02-04

**Authors:** Yinjie Su, Weilian Feng, Juanyi Shi, Luping Chen, Jian Huang, Tianxin Lin

**Affiliations:** 1 The Department of Urology, Sun Yat-Sen Memorial Hospital, Sun Yat-Sen University, Guangzhou, China; 2 The Department of Endocrinology, Sun Yat-Sen Memorial Hospital, Sun Yat-Sen University, Guangzhou, China; 3 The Department of Pediatric Surgery, Sun Yat-Sen Memorial Hospital, Sun Yat-Sen University, Guangzhou, China

**Keywords:** Bladder cancer, circRIP2, EMT, miR-1305, Tgf-β2

## Abstract

**Background:**

Increasing evidences indicate that circular RNAs exert critical function in regulating bladder cancer progression. However, the expressive patterns and roles of circular RNAs in bladder cancer remain less investigated.

**Methods:**

circRIP2 was identified and evaluated by RNA-sequencing and qPCR; in vitro effects of circRIP2 were determined by CCK8, clone forming, wound healing and trans-well assays; while mice subcutaneous tumor model was designed for in vivo analysis. Western blot, RNA pulldown assay, miRNA capture and dual luciferase assessment were applied for mechanistic studies.

**Results:**

circRIP2 was identified as a conserved and dramatically repressed circular RNA in bladder cancer. Patients that displayed higher circRIP2 expression negatively associate with the grade, stage, metastasis as well as outcome of bladder cancer. In vitro and in vivo studies suggest that circRIP2 enables to promote bladder cancer progression via inducing EMT. Regarding the mechanism, we performed RNA-sequencing analysis, RNA pulldown with biotin-labeled circRIP2-specific probe, dual luciferase reporter assay. It was found that circRIP2 enables to sponge miR-1305 to elevate Tgf-β2 in bladder cancer, and inducing EMT via Tgf-β2/smad3 pathway. Blocking Tgf-β2 in bladder cancer deprives circRIP2 induced cancer progression and EMT.

**Conclusions:**

Taken together, our study provides the first evidence that circRIP2 expresses differentially in bladder cancer and negatively along with the cancer progression; effective circRIP2 activity accelerates bladder cancer progression via inducing EMT by activating miR-1305/Tgf-β2/smad3 pathway. The research implies that circRIP2 might be a potential biomarker and therapeutic target for bladder cancer patients.

## Introduction

The risk of bladder cancer in affecting men’s health ranks the top one in Chinese urological cancer [1]. Surgical resection and chemotherapy are two mainstreams for bladder cancer treatment. However, the effect is not satisfactory when cancer has developed into advanced stage which presented as easily distant metastasis, devastatingly drug resistance and poor clinical outcome [2]. Because of lack of diagnostic and specifically therapeutic approaches, most patients are diagnosed as high grade tumor as soon as they are found [3]. Facing with those urgent challenge, it is of vital importance to explore novel biomarker and identify effective therapeutic targets for bladder cancer.

circular RNAs are a new class of spliced RNA form and have been identified to play an ultimate role in cancer biology. Considering its covalently closed loop without 5′-3′ ployadenylation and polar tails, circular RNAs extremely resist multiples exonuclease and display an extraordinarily stable state that suggest its potential as a biomarker [4]. Hence, researches of circular RNAs are divided into 2 parts, one is to identify its carcinogenic role, the other is to prove its application as a biomarker [5]. Serving as an oncogene or tumor suppressor gene, in bladder cancer, ciRS-7 is found to suppress bladder cancer growth by elevating p21 [6]; hypoxia elevates circELP3 to promote bladder cancer progression and drug resistance [7]; circular RNA CEP128 promotes bladder cancer cell propagation and migration via regulating MAPK signaling [8]; and circHIPK3 decreases lung metastasis through suppressing heparanase expression [9]. Besides, as a biomarker [10], up-regulated circPRMT5 in the exosomes from serum and urine positively correlates with metastasis of bladder cancer patients [11]. circ-ITCH [12] and circHIPK3 [9] express lower in bladder cancer tissues, and negatively associate with grade, stage as well as lymph node metastasis of bladder cancer patients. Taken together, these results imply a significant role of circular RNA in regulating bladder cancer behaviors and serves as a novel biomarker for bladder cancer.

To identify more circular RNAs in bladder cancer, we re-analyzed our previous RNA-sequence in 2 paired bladder cancer tissues [7]. Among various circular RNAs that specifically reduced in bladder cancer, circRIP2 ranks the top 3. It was found that circRIP2 significantly down-regulated in bladder cancer tissues, and negatively associated with bladder cancer grade, stage, metastasis as well as better patients’ outcome. In vitro and in vivo studies suggest that circRIP2 promotes bladder cancer proliferation, invasion and migration via stimulating EMT through Tgf-β2/smad3 pathway. In a word, our study provides the first evidence that circRIP2 expresses differentially in bladder cancer and negatively predicts cancer progression; effective circRIP2 activity accelerates bladder cancer progression via inducing EMT by activating miR-1305/Tgf-β2/smad3 pathway. The research implies that circRIP2 may be a potential biomarker and therapeutic target for bladder cancer patients.

## Materials and methods

### Human tissues management

Forty-five paired bladder cancer and the adjacent normal tissues, 58 bladder cancer tissues were collected from Sun Yat-Sen Memorial Hospital from 2014 Jun 1st to 2019 Mar 1st. All studies were permitted by The Ethics Committee of Sun Yat-Sen University. All patients signed the contract to use their tissues experimentally. 3 pathologists confirmed the pathologic and histological diagnosis. All patients were followed till died.

### Cell culture

Two bladder cancer cell lines 5637 (RRID: CVCL_0126) and UM-UC-3 (U3, RRID: CVCL_1783) were purchased from ATCC. U3 was cultured in DMEM medium (Gibco, USA) and 5637 was in 1640 medium (Gibco, USA) respectively, with 100 U/ml penicillin and streptomycin (Gibco, USA) and 10% FBS (Gibco, USA). Cells were cultured in humidified, constant 37 °C and 5% CO_2_ incubator (Thermo, Germany). All cell lines were validated by IGE BIOTECHNOLOGY LTD (Guangzhou, China) with STR assessments in recent 6 months. 

### RNA preparation and quantitative real-time PCR

RNA from cell or tissue samples was extracted by a rapid RNA extraction kit (ES Science, China) and reverse transcribed by PrimerScript™ RT Master Mix Kit (TakaRa, Japan). RNA expression was evaluated by quantitative real-time PCR that was applied by TB Green Premix Ex Taq II Kit (TakaRa, Japan). All primers were listed in Additional file 6: Table S1.

### Agarose gel electrophoresis and RNAse R treatment

Detailed procedures were shown in our previously published research [7].

### Florescent in situ hybridization (FISH)

FISH probes for circRIP2 and miR-1305 were synthesized by Genepharm (Suzhou, China). FISH assay was performed according to the kit of “Florescent *in situ* hybridization” from RiboBio (Guangzhou, China). 18SRNA was taken as positive control.

### Nuclear and cytoplasmic extraction assay

To extract nuclear and cytoplasmic RNA, kit of ThermoFisher (78,833, German) was applied.

### Cell transfection

siRNAs to target circRIP2 and RIP2 were purchased from Genepharm (Suzhou, China). Transfection was performed by lipofectamine iMAX (Gibico, USA). Sequence of siRNAs were listed in Additional file 6: Table S2.

Stable overexpressed circRIP2 cells were constructed with vector “plenty-ciR-GFP-T2A-puro” that was synthesized by IGE BIOTECHNOLOGY LTD (Guangzhou, China).

### CCK8 viability assay

1500 cells/well were seeded in 96-well plate 24 h before. 100 μl culture medium that contained 10% CCK8 (Beyotime, Suzhou, China) was incubated in each well for 2 h. OD values under 452 nm were measured by microplate reader (TECAN Spark 10 M, Shengyang, China).

### Clone formation assay

1500 cells/well were seeded in 6-well plate. Clones were harvested when over 50 cells per clone were counted. Clones were stained with 1% crystal violet.

### Trans-well for migration and matrigel invasion assay

600 μl culture medium with 10% FBS was added in lower chamber. Single cell suspension with 80,000 cells was seeded on upper chamber in 200ul non-serum culture medium for cell migration and 200ul matrix gel for cell invasion; after being incubated 24 h for U3 and 38 h for 5637 cells, upper chamber cells were fixed with 4% paraformaldehyde for 5 min and stained with 0.1% crystal violet for another 5 min. Cells that passed through membrane were counted under light microscope (Nikon, Ni-U, Japan).

### Wound healing assay

Cells in 100% density were seeded on 6-well plate 12 h before and scratched with 200ul pipette tips to create a wound. The ability of wound healing was measured by distance between two sides of the induced injury. Scratched lines were photographed under 0 h and 24 h.

### Tumor subcutaneous mice model

All studies involved in mice model were permitted by “The Animal Management Committee” of Sun Yat-Sen University. 10^7^ cells were subcutaneously injected in nude mice (3–4 weeks old, male). Volume (V) and weight (W) were measured twice a week. Mice were sacrificed when tumor volume reached over 200mm^3^. V = (L × W × W)/2, weight (W) and length (L).

### Western blot

Antibodies for E-cadherin, N-cadherin, Vimentin, Tgf-β2, smad2, smad3, p-smad2, p-smad3 were purchased from Santa Cruze (USA) and diluted in 1:1000 respectively.

### Dual luciferase reporter assay

Predicted binding sites between circRIP2 and miR-1305, miR-1305 and Tgf-β2 were cloned inside a dual luciferase reporter vector which named psi-check2 by Synbio Technologies (Suzhou, China). Dual luciferase reporter vector and miR-1305 mimics were co-transfected into 293 T cells. Luciferase activity was measured 48 h later according to manufacture’s procedures (E292, Promega, USA).

### RNA pulldown

Detailed procedures were described in our previous paper. Specific biotin-labeled probes for circRIP2 and biotin labeled miRNA 653-5p mimics was designed and synthesized by Genepharm (Suzhou, China). Sequence of each probe in this study was listed in Additional file 6: Table S3.

### Statistical analysis

All results in this paper were statistically analyzed by GraphPad 5.0. T-test was performed between 2 independent group; one-way ANOVA test was applied among various group; Kaplan-Meier curves and log-rank test analyze patients’ survival. *P* < 0.05 was thought as statistical significance.

## Results

### Higher circRIP2 level associates with better bladder cancer outcome

**Fig. 1:**
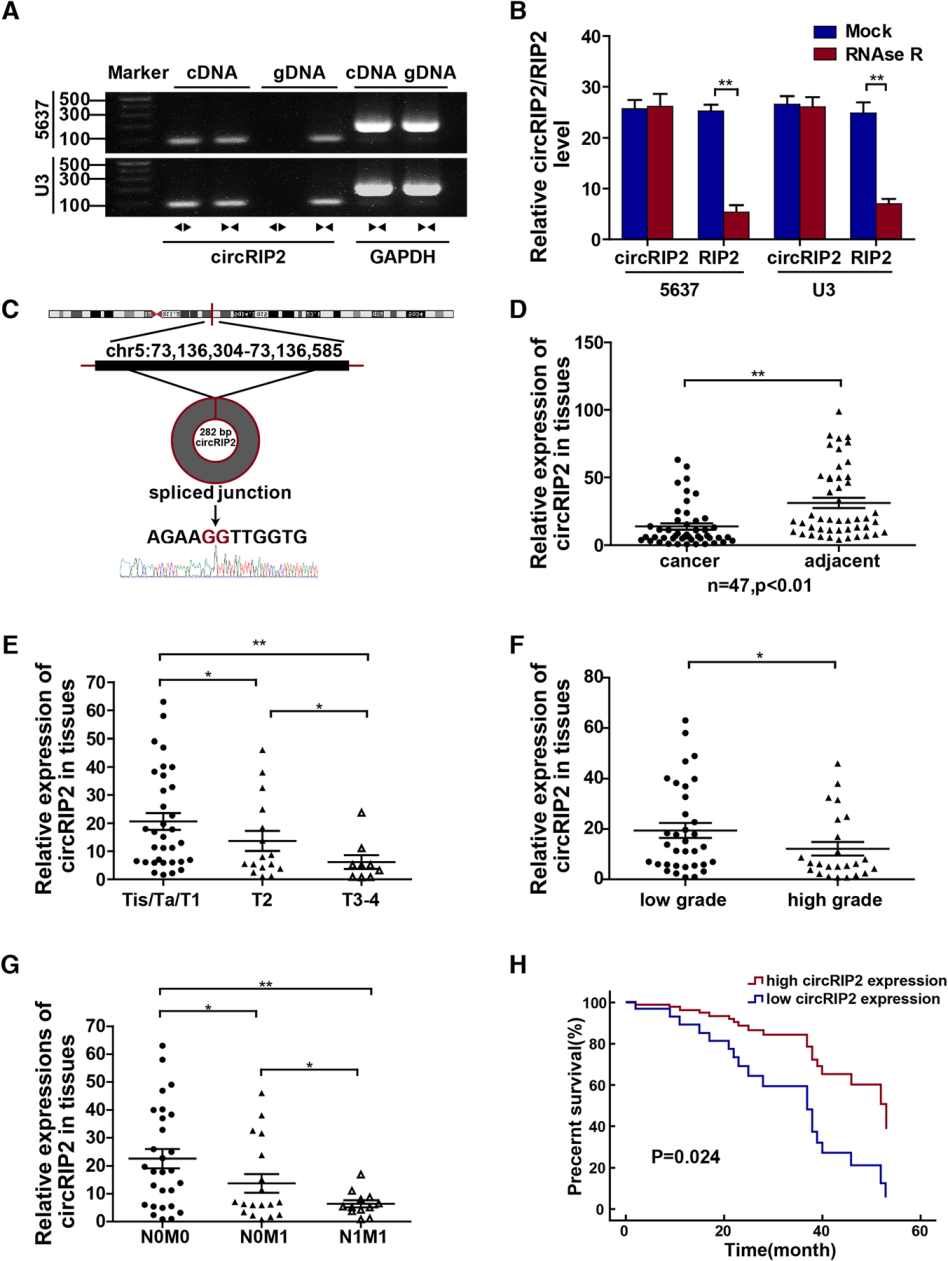
Higher circRIP2 level in bladder cancer associates with better outcome. Divergent (circRIP2, ◀▶) and convergent (RIP2, ▶◀) primers were designed; **a** To detect circRIP2 is circular RNA, agarose gel electrophoresis showed that circRIP2 was amplified by divergent primer in cDNA but not in gDNA. GAPDH was taken as negative control; **b** RNAse R treatment assay was performed, and only circRIP2 showed strong exonuclease resistance but not the RIP2; **c** sanger sequence of circRIP2; **d** qPCR was used to evaluate the expression of circRIP2 in 45 paired bladder cancer tissues; **e** circRIP2 level among 58 bladder cancer tissues was analyzed by T stages; **f** circRIP2 level among 58 bladder cancer tissues was analyzed by tumor grade; **g** circRIP2 level among 58 bladder cancer tissues was analyzed by metastasis; **h** circRIP2 level among 43 patients in following was analyzed for overall survival.

To first identify the molecule we were studying is circular RNA, we performed agarose gel electrophoresis and RNAse R treatment. It was shown that circRIP2 lost apparently in gDNA (Fig. 1a) but remained more stable than RIP2 after RNAse R treatment (Fig. 1b). Sanger sequence showed that circRIP2 was same as has_circ_0005777 that is searched from www.circbase.com (Fig. 1c). These results suggested that circRIP2 is circular RNA. Next, to analysis the clinical value of circRIP2 in bladder cancer, level of circRIP2 in 58 bladder cancer tissues, 45 paired bladder cancer tissues and the adjacent normal tissues was detected and analyzed. It’s found that circRIP2 reduces 2.28 folds in bladder cancer tissues when compared with the adjacent normal tissue (Fig. 1d); expression of circRIP2 negatively correlates with development of bladder cancer, as demonstrated by higher circRIP2 level among lower T stage (Fig. 1e), tumor grade (Fig. 1f) and less metastatic patients (Fig. 1g). Additionally, to assess whether the expression level of circRIP2 can be used as an independent prognostic predictor of bladder cancer patients, univariate and multivariate Cox hazard analysis were performed to assess the relationship between clinical pathological parameters, including the expression of circRIP2 and the prognosis of the patients with bladder cancer (Table 1). It suggests that higher circRIP2 level indicates better patients’ outcome (Fig. 1h), with (Hazard ratio [HR] = 0.332; 95% CI: 0.122–0.904, *P* = 0.031). The results imply that circRIP2 expresses lower and displays a tumor suppressive role in bladder cancer. Relationship between circRIP2 level and multiple clinical parameters was concluded in Table 2.

**Table 1:** Univariate and multivariate analysis of overall survival

Variable	Univariate analysis			Multivariate analysis		
	HR	95%CI	*p* value	HR	95%CI	*p* value
Age (≤53.21 VS > 53.21)	2.915	1.153–7.374	0.024^*^			
Gender (Male VS Female)	1.284	0.443–3.720	0.645			
T stage (<=T1 VS > T2)	14.701	3.349–64.533	< 0.001^*^	15.397	3.435–69.005	< 0.001^*^
Lymph node metastasis (N0 VS N1–2)	6.791	1.557–29.622	0.011^*^			
Distant metastasis (M0 VS M1)	4.88	1.914–12.437	0.001^*^			
circRIP2 expression (High VS Low)	0.339	0.128–0.898	0.029^*^	0.332	0.122–0.904	0.031^*^

*HR*: Hazard ratio; *95% CI*: 95% confidence interval.

*p < 0.05 represents statistical significance

**Table 2:** Relationship between circRIP2 level and clinical characteristics in bladder cancer

Total	Patients	Expression of circRIP2		
		High	Low	p
Age (mean)	53.21	51.32	50.27	0.713
Gender				
Male	46	20	26	0.614
Female	12	5	7	
Tumor stage				
Tis/Ta/T1	33	13	20	0.038
T2	16	8	8	
T3/T4	9	4	5	
Grade				
High	34	9	25	0.002
Low	24	16	8	
Number of tumors				
Solitary	41	22	19	0.584
Multiple	17	3	14	
Metastasis				
N0 M0	28	17	11	0.012
N1 M0	18	8	10	0.009
N1 M1	12	0	12	
Follow-up(month, mean)	33.23	31.83	33.67	0.024^a^

*p* < 0.05 represents statistical significance (Chi-square test)

^a^ Results are analyzed by Kaplan-Meier curves and log-rank test

### circRIP2 promotes bladder cancer cells’ progression

**Fig. 2:**
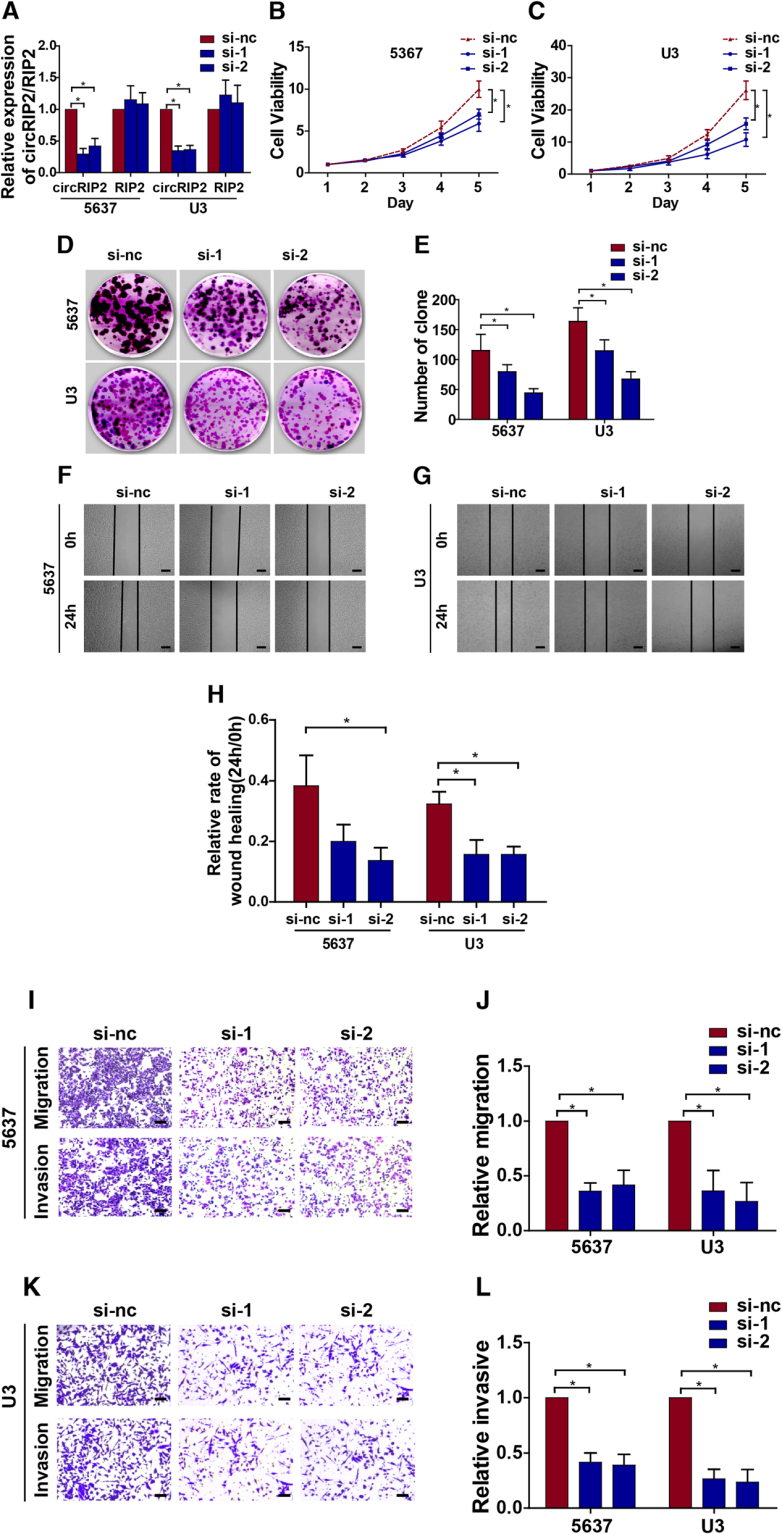
Silencing circRIP2 suppresses bladder cancer progression. circRIP2 silencing was performed by siRNAs interfering. **a** Level of circRIP2 in 5637 and U3 cells after siRNAs silencing were detected by qPCR. nc was taken as normal control; **b**, **c** CCK8 assay was performed to detect cell viability of bladder cancer cells; **d**, **e**. Cell potential to replicate and self-renew was reflected by clone formation; **f**, **g**, **h** Wound healing assay showed cell potential of migration; scale bar: 100 μm; **i**, **j**, **k**, **l**. Trans-well for migration and matrigel invasion assay showed cell potential of migration and invasion; scale bar: 25 μm.

**Fig. 3:**
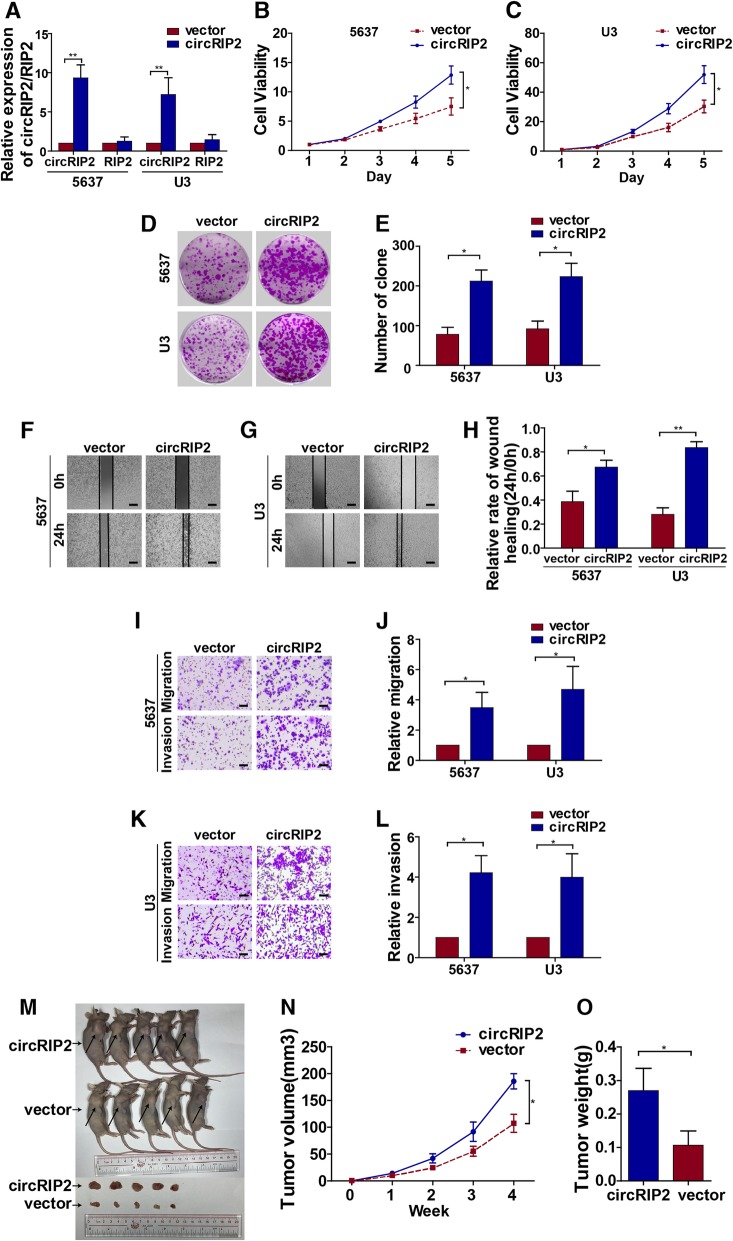
Over-expressing circRIP2 promotes bladder cancer progression. Bladder cancer cells that stably over-expressed circRIP2 was constructed. **a** Level of circRIP2 in 5637 and U3 cells after circRIP2 overexpression were detected by qPCR. Vector was taken as normal control; **b**, **c**. CCK8 assay was performed to detect cell viability of bladder cancer cells; **d**, **e**. Cell potential to replicate and self-renew were reflected by clone formation assay; **f**, **g**, **h**. Rate of wound healing assay showed cell potential of migration; scale bar: 100 μm; **i**, **j**, **k**, **l** Trans-well migration and matrigel invasion assay showed cell potential of migration and invasion; scale bar: 25 μm; **m**, **n**, **o** Over-expressed circRIP2 bladder cancer cells were injected subcutaneously to detect in vivo effect of circRIP2. *n* = 5 mice for each group.

To next evaluate effect of circRIP2 on bladder cancer cells' behaviors, 2 siRNAs were synthesized and the interfering efficiency was evaluated by qPCR (Fig. 2a). Cell growth responds directly to different interferences. Hence, we first checked the effect of circRIP2 on cell viability. Silencing circRIP2 dramatically reduced cell viability of bladder cancer (Fig. 2b c). Cell growth relies on a harmonious balance between proliferation and apoptosis. To identify whether this growth reduction caused by circRIP2 silencing was contributed by an increased apoptosis or reduced cell proliferation, we subsequently performed Annexin V/Pi apoptotic assay and clone formation assay. It was found that silencing circRIP2 leads less effect on cell apoptosis (Additional file 1: Figure S1), but significantly represses clone formation of bladder cancer cells (Fig. 2d, e). The results suggest that silencing circRIP2 decreases bladder cancer growth in vitro. Besides proliferation, metastasis is another essential for cancer progression. Next, to determine the role of circRIP2 in cancer metastasis, trans-well and wound healing assay were performed. It was found that silencing circRIP2 apparently reduced wound healing capability of bladder cancer cells (Fig. 2f, g, h), as well as the migration and invasion potential (Fig. 2i, j, k, l). The results suggest that silencing circRIP2 suppresses bladder cancer metastasis in vitro. Furthermore, bladder cancer cells that over-expressed circRIP2 were constructed as well (Fig. 3a). Opposite from circRIP2 silencing, overexpressed circRIP2 in bladder cancer promoted cells viability (Fig. 3b, c), increased clone formation (Fig. 3d, e), encouraged cell invasion and migration (Fig. 3i, j, k, l) and accelerated wound healing potential of bladder cancer cells (Fig. 3f, g, h). Above all, the results indicate that circRIP2 promotes proliferation and metastasis of bladder cancer cells in vitro. Additionally, to assess in vivo effect of circRIP2 on tumorigenicity, a subcutaneous tumor mice model was applied. Overexpressed circRIP2 significantly promotes bladder cancer growth in nude mice (Fig. 3m, n, o). Taken together, the above results suggest that circRIP2 accelerates bladder cancer cells’ progression.

### circRIP2 sponges miR-1305 to elevate Tgf-β2

**Fig. 4:**
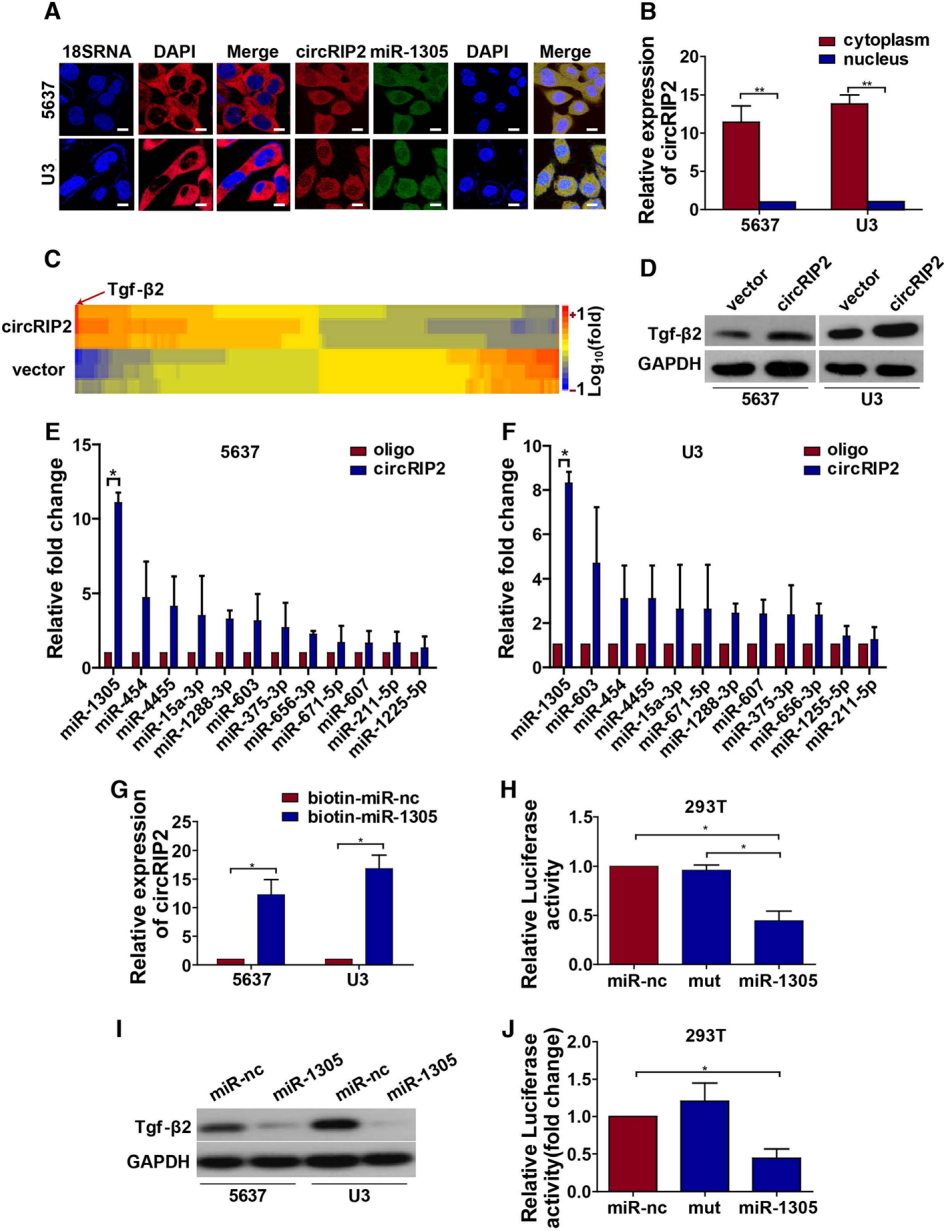
circRIP2 sponges miR-1305 to elevate Tgf-β2 in bladder cancer cell. **a**, **b** FISH and nuclear-plasma extraction assay were performed to detect the location of circRIP2. Cytoplasm located 18SRNA was taken as positive control, scale bar: 2.5 μm; **c**. Heat map and RNA-sequencing analyze mRNAs expression that were differently expressed between circRIP2 over-expressed and vector in U3 bladder cancer cells; Each group contains 3 samples; Each column corresponds to the expression profile of a sample, and each row represents a mRNA (log fold change (FC) ≥1.5 and *P* < 0.05); **d**. Western blot re-verified the up-regulation of Tgf-β2 caused by circRIP2 over-expression; **e**, **f**. RNA pull-down assay was used to evaluate miRNAs that could bind with circRIP2 in 2 bladder cancer cells, 5637 and U3; **g**. Pull-down assay for biotin labeled miRNA was used to evaluate binding properties between miR-1305 and circRIP2 in 2 bladder cancer cells, 5637 and U3; **h**. Dual luciferase reporter assay was used to prove the binding properties between circRIP2 and miR-1305; **i**. Western blot showed a significant interfering effect on Tgf-β2 expression by miR-1305; **j**. Dual luciferase reporter assay showed the binding property between miR-1305 and Tgf-β2;

To further identify the underlying mechanism, we analyzed the differentially expressed genes after circRIP2 over-expression via RNA sequencing. It was found that Tgf-β2 up-regulated the most among various altered genes (Fig. 4c) which was furtherly verified by western blot (Fig. 4d). We wonder whether effect of circRIP2 is processed by Tgf-β2 elevation. We added Tgf-β2 inhibitor in circRIP2 over-expressed cells. It was found that compared with circRIP2 over-expression alone, Tgf-β2 inhibitor significantly deprived tumor promotive effect of circRIP2, as demonstrated by reduced cell viability, clone formation, wound healing, cell invasion and metastasis (Additional file 2: Figure S2). The results indicate that circRIP2 promotes bladder cancer progression via elevating Tgf-β2. We next investigated how could circRIP2 up-regulates Tgf-β2? We performed FISH (Fig. 4a) and nuclear-plasma extraction assay (Fig. 4b). It was found that circRIP2 mainly exists in cytoplasm which suggests a miRNA sponging role here. With bio-imformatic prediction from 3 websites, including Interactome, circBank and circMIR, 6 miRNAs from 12 overlapped miRNAs were dragged commonly in both 2 bladder cancer cells by RNA pulldown assay (Additional file 3: Figure S3). Among these 6 miRNAs, miR-1305 changed the most. (Fig. 4e, f). Besides, Tgf-β2 is the direct downstream target of miR-1305 that is predicted from TargetScan and miRDB together. These results suggest that circRIP2 might sponge miR-1305 to elevate Tgf-β2. Hence, we then designed biotin-labeled miR-1305, as expected, circRIP2 was significantly collected by miR-1305(Fig. 4g); and transferring miR-1305 and a dual-luciferase reporter that contained circRIP2 binding sites into 293 T cells together, a dramatic reduction of luciferase activity was observed (Fig. 4h). In addition, level of neither miR-1305 nor circRIP2 were changed when each of them was over-expressed (Additional file 4: Figure S4). These results suggest that circRIP2 sponges miR-1305. To next determine the binding property between miR-1305 and Tgf-β2, western blot (Fig. 4i) and dual-luciferase reporter assay (Fig. 4j) demonstrated that miR-1305 significantly reduced expression and luciferase activity of Tgf-β2. All in all, the results above suggest that circRIP2 sponges miR-1305 to elevate Tgf-β2 in bladder cancer cells.

### miR-1305 rescues the tumor progressive role of circRIP2

**Fig. 5:**
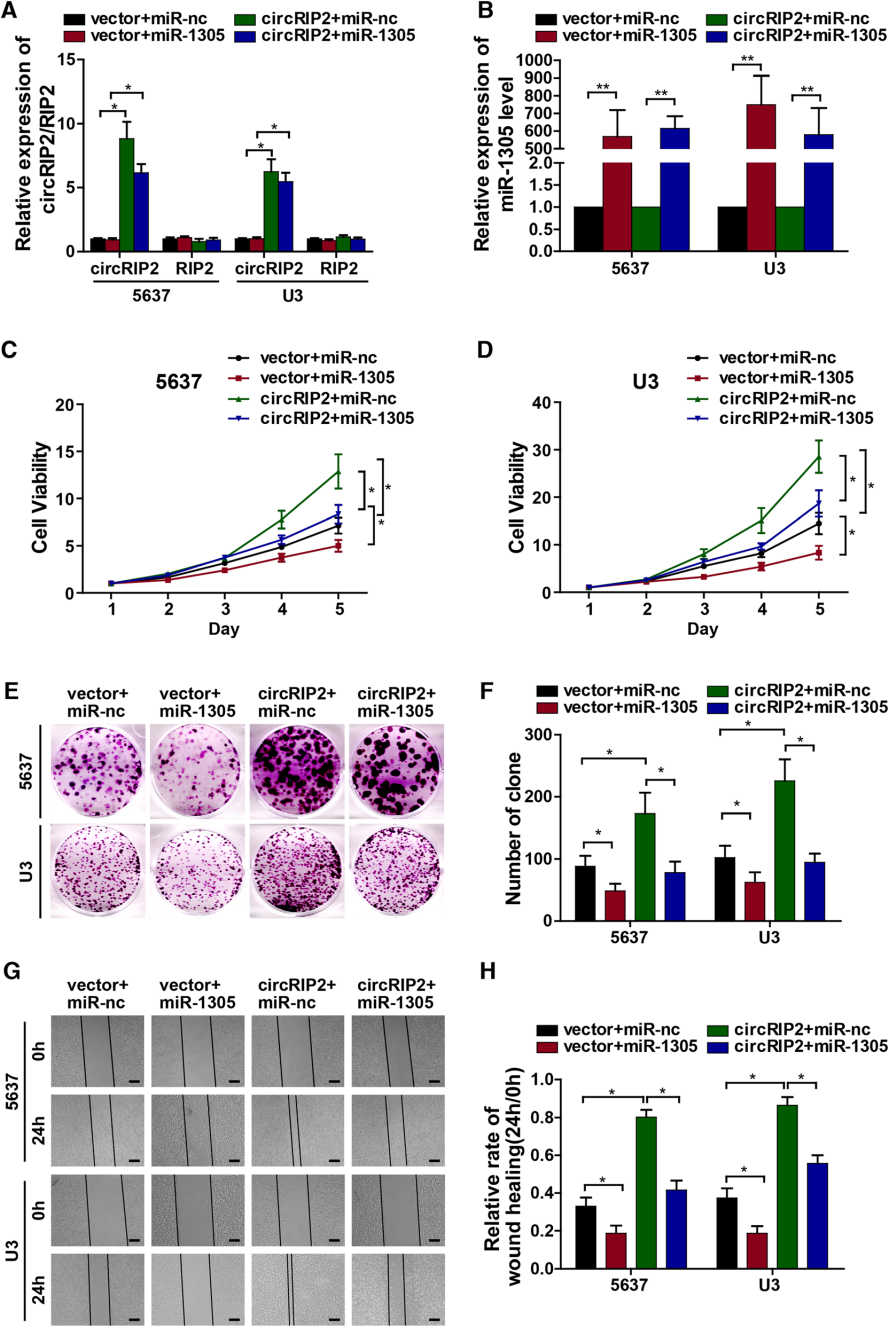
miR-1305 rescues the tumor progressive role of circRIP2. To prove tumor suppressive role of circRIP2 could be rescued by miR-1305. miR-1305 and miR-nc were introduced together into circRIP2 over-expressed and control cells. **a**, **b**. qPCR was taken to evaluate circRIP2, RIP2, miR-1305 expression; **c**, **d**. CCK8 assay was performed to detect cell viability of bladder cancer cells; **e**, **f**. Cell potential to replicate and self-renew was reflected by clone formation; **g**, **h**. Rate of wound healing assay showed cell potential of migration; scale bar: 100 μm. **i**, **j**, **k**, **l**, **m**. Trans-well migration and matrigel invasion assay showed cell potential of migration and invasion of bladder cancer cells; scale bar: 25 μm.

We have proved that circRIP2 promotes bladder cancer progression via elevating Tgf-β2; and circRIP2 sponges miR-1305 to elevate Tgf-β2 in bladder cancer cells. To further prove that the tumor progressive role of circRIP2 was exerted via sponging miR-1305, several miRNA rescue experiments was applied. We first checked the role of miR-1305 in bladder cancer. It was found that miR-1305 significantly suppressed bladder cancer proliferation, with regard on decreased cell viability (Fig. 5c, d), clone formation (Fig. 5e, f); wound healing (Fig. 5g, h), cell invasion and metastasis (Fig. 5i, j, k, l, m). Next, we introduced circRIP2 and miR-1305 together into bladder cancer cells. It was shown that exogenous miR-1305 over-expression significantly deprived tumor progressive role of circRIP2. This effect was demonstrated by decreased cell viability (Fig. 5c, d), clone formation (Fig. 5e, f); wound healing (Fig. 5g, h), cell invasion and metastasis (Fig. 5i, j, k, l, m) when compared with circRIP2 overexpression alone. Taken together, the above results suggest that miR-1305 rescues the tumor progressive role of circRIP2.

### circRIP2 stimulates EMT via Tgf-β2/smad3 pathway

**Fig. 6:**
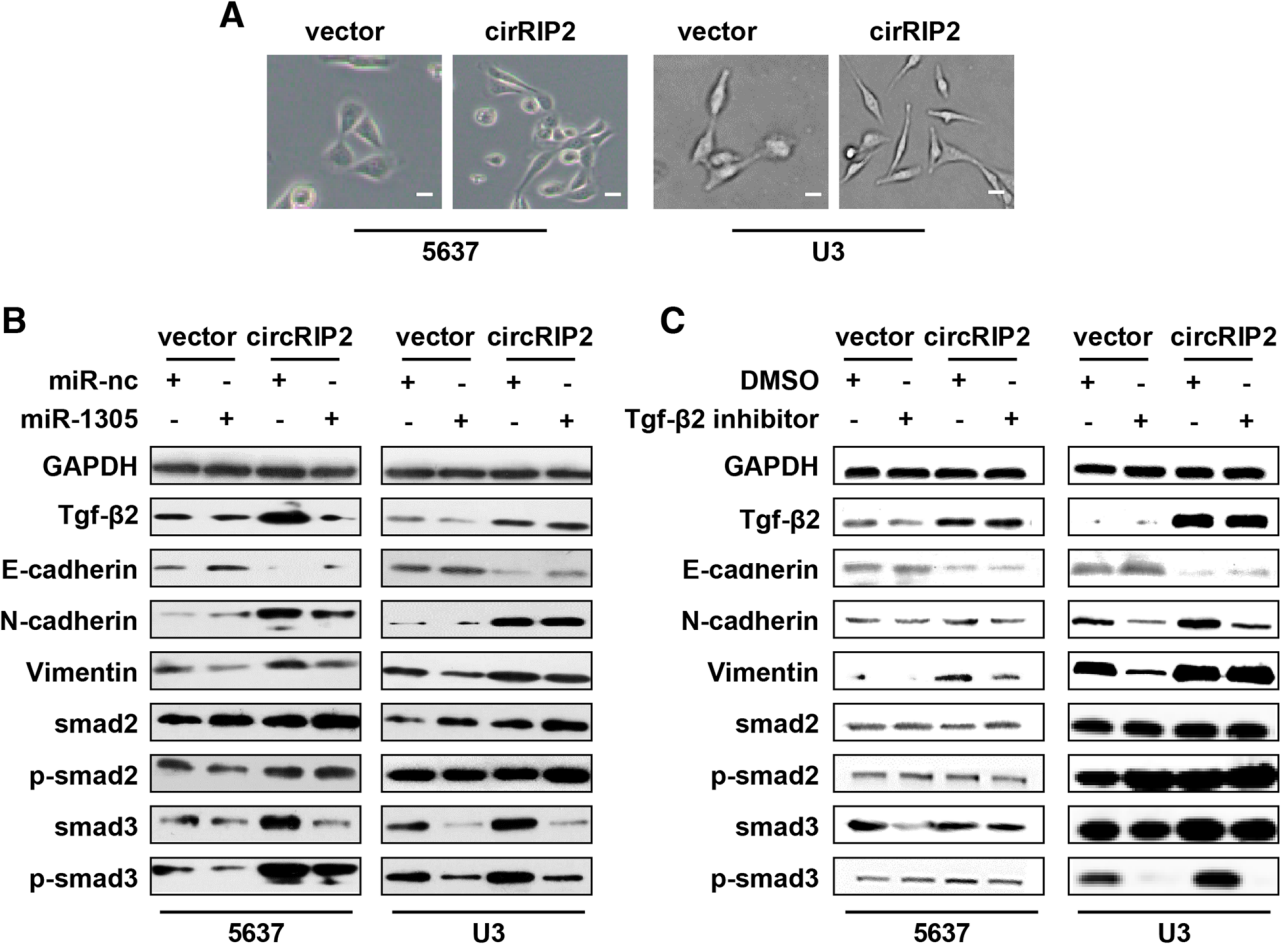
circRIP2 contributes to EMT via Tgf-β2/smad3 pathway. **a**. Cell morphology of bladder cancer cells in vector and circRIP2 overexpressed cells was observed under light microscope (400×); **b**, **c**. Western blot showed expression of different epithelial and mesenchymal markers.

Tgf-β signaling plays an ultimate role in cancer progression [13]; enhanced Tgf-β signaling in cancer accelerates EMT and maintained a highly proliferative phenotype [14–17]. As circRIP2 elevates Tgf-β2 in bladder cancer, it is interesting to investigate whether circRIP2 enables to lead to EMT as well as accelerates bladder cancer progression. As expected, cells that over-expressed circRIP2 displayed a spindle-like structure when compared with a sharp edge on the vector (Fig. 6a). Moreover, western blot revealed that expression of E-cadherin, an epithelial marker, was decreased; whereas, expression of Tgf-β2, N-cadherin and Vimentin, which are mesenchymal markers, were alternatively increased after circRIP2 over-expression (Fig. 6b). These results suggest that circRIP2 induces EMT in bladder cancer. As we mentioned before, circRIP2 sponges miR-1305 to elevate Tgf-β2 in bladder cancer cells. We then overexpressed circRIP2 and miR-1305 together, and a deprivation of circRIP2 induced mesenchymal phenotype was found (Fig. 6b), as well as by Tgf-β2 inhibition (Fig. 6c). These results suggest that circRIP2 contributes to EMT via sponging miR-1305 and elevating Tgf-β2. Effective Tgf-β signaling needs smad pathway. Activated smad2/smad3/smad4 complex by Tgf-β stimulation constitutes the final transcriptional regulation of Tgf-β signaling. As circRIP2 induces EMT via Tgf-β2, we examined expression of smad2, p-smad2, smad3 and p-smad3. It was found that circRIP2 significantly elevated smad3 and p-smad3 level, but exerted less effect on smad2 and p-smad2; overexpressing circRIP2 and miR-1305 together deprived smad3 and p-smad3 elevation in circRIP2 over-expressed cells (Fig. 6b); inhibiting Tgf-β2 repressed smad3 and p-smad3 activation (Fig. 6c). Hence, these results suggest that circRIP2 stimulates EMT via Tgf-β2/smad3 pathway.

## Discussion

Circular RNAs are gradually defined to be ultimate in bladder cancer [5, 18, 19]. Because of its extremely stable circular structure, much emphasis was put on its application as biomarker. For example, a positive correlation was analyzed between serum circPRMT5 and metastasis of bladder cancer [11]; negative associations among circ-ITCH [12], circHIPK3 [9] and cancer grade, stage as well as lymph node metastasis of bladder cancer were identified gradually. However, as less cohorts were involved in these studies, future researches expect larger enrolled individuals, not only from patients’ tissues but from patients’ serum or urine, to further analyze the potential of circular RNAs as biomarkers for bladder cancer. Additionally, except for the role as promising biomarker, circular RNAs were also proved to work as oncogenes or tumor suppressor genes in bladder cancer. Such as, ciRS-7 enables to suppress cancer growth by elevating p21 [6]; circELP3 promotes progression and drug resistance of bladder cancer [7]; circHIPK3 decreases lung metastasis through suppressing heparanase expression [9]; circular RNA CEP128 promotes bladder cancer cell propagation and migration via MAPK signaling [8]. Nevertheless, the mechanisms of circular RNAs in bladder cancer are far from being well identified.

In this study, we demonstrated that circRIP2 ables to promote bladder cancer progression via inducing EMT by activating miR-1305/Tgf-β2/smad3 pathway. EMT is a common phenomenon in epithelial-derived malignant tumors and participates ultimately in progression, especially invasion and treatment tolerance of malignant tumor [14–17]. Tgf-β signaling orchestrates an intricate signaling network to modulate tumorigenesis and cancer progression via EMT. The Tgf-β family include: Tgf-β1, Tgf-β2, Tgf-β3. When activated, these ligands bind to type II receptor, which recruits and phosphorylates type I receptor. The type I receptor then phosphorylates receptor-regulated SMADs (R-SMADs: smad2/3) which can bind the coSMAD (smad4). R-SMAD/coSMAD complex accumulates in nucleus where they acts as transcription factor and participates in the regulation of target genes’ expression [20]. Although the downstream signaling of Tgf-β signaling has been fully identified, mechanisms of how Tgf-β family are regulated remain less investigated. Here, we firstly proved that Tgf-β2 could be elevated by circRIP2 in bladder cancer. Up-regulation of circRIP2 encourages bladder cancer via Tgf-β2 induced EMT; inhibiting Tgf-β2 significantly deprives circRIP2 mediated bladder cancer progression. Taken together, our study firstly proves that circRIP2 enables to regulate Tgf-β pathway and promotes bladder cancer progression; besides, the research also suggests a new therapeutic target and biomarker for bladder cancer.

However, what is interesting is that, circRIP2 serves as a tumor suppressor in clinical level, but the majority of our results demonstrate a cancer promotive role of circRIP2 in vitro and in vivo. This inconsistency between clinic and biological level suggests that there could be more molecular mechanisms for circRIP2 in regulating bladder cancer behaviors. For example, except for the regulation on tumor itself, circRIP2 might in some extent affect tumor micro-environment, and the final result displays a tumor suppression. To stand our hypothesis, we firstly checked our RNA-sequences when circRIP2 was over-expressed. It was found that except for Tgf-β signaling, chemokines (CCL2, CCL3, CXCL5, CXCL17 and CXCL20) and cytokines (IL-6, IL-13 and IL-17) that especially communicate with immune cells were also changed dramatically. Besides, to further prove circRIP2’s role in driving bladder cancer micro-environment, we evaluated immune states in bladder cancer patients’ tissues, which are based on CD3 and CD8 cells infiltration. As expected, in higher circRIP2 expressed patients, more CD3^+^/CD8^+^ cells were identified when compared with lower circRIP2 expression (Additional file 5: Figure S5). These findings suggest that the oncogenic circRIP2 may play a tumor suppressive role by communicating with the micro-environment comprehensively. Additionally, facing with such inconsistency, several studies also support a tumor suppressive role of oncogenes which focused more on the tumor micro-environment. For example, miR-21 is an oncogene. However, knocking out miR-21 in mice weakened proliferation of both CD4+ and CD8+ cells, reduced cytokine production and accelerated growth of grafted tumor via Pten/Akt pathway [21]. Lats1/2 were considered as tumor suppressor. Loss of Lats1/2 promotes tumor cell growth in vitro, but inhibits tumor growth in murine tumor models. Moreover, impairing Lats1/2 function in tumors improves immunogenicity via inducing type I interferon response through Toll-like receptors-MYD88/TRIF pathway [22, 23]. Taken together, these results suggest that it is necessary to consider the involvement of tumor micro-environment if we want to comprehensively define a gene’s role.

Although we firstly proved that circRIP2 promotes bladder cancer progression via inducing EMT, the exact role about how circRIP2 represses cancer progression through regulating cancer immunity remains poorly understood. Hence, future studies are urgent to fully characterize the underlying mechanisms of circRIP2 in bladder cancer as we can safely and efficaciously target this kind of RNA in patients.

In a word, the findings expand our current knowledge of circular RNAs in cancer and indicate a promising biomarker and therapeutic target by focusing on circRIP2 in bladder cancer.

## Supplementary information

**Additional file 1: Figure S1.** Silencing circRIP2 contributes less effect on bladder cancer apoptosis. A.B. Annexin V/Pi apoptotic assay was performed to detect effect of circRIP2 on the apoptosis of bladder cancer cells.

**Additional file 2: Figure S2.** Tgf-β2 inhibitor revered cancer promotive role of circRIP2 in bladder cancer cells in vitro. A,B. CCK8 assay was performed to detect cell viability of bladder cancer cells; C,D. Cell potential to replicate and self-renew was reflected by clone formation; E,F. Rate of wound healing assay showed cell potential of migration; scale bar: 100μm. G,H,I,J,K. Trans-well migration and matrigel invasion assay showed cell potential of migration and invasion of bladder cancer cells; scale bar: 25μm.

**Additional file 3: Figure S3.** miRNAs that may bind with circRIP2 was predicted. 12 overlapped miRNAs were predicted from Interactome, circbank and circMIR together.

**Additional file 4: Figure S4.** Expression of miR-1305 and circRIP2 was detected under each over-expression. A.B qPCR was used to detect expression of circRIP2 or miR-1305.

**Additional file 5: Figure S5.** Higher circRIP2 patients display stronger immune infiltration. A,B Immune histochemistry detected infiltration of CD3 and CD8 cells among paraffin-embedded tissues. Cells in 10 randomly selected views were counted. Views were photographed under 200× microscopically.

**Additional file 6: Table S1.** List of primers for qPCR. **Table S2.** List of sequences for siRNAs. **Table S3.** List of probe sequences for RNA pulldown.

## Data Availability

From 2014 Jun ^1^st to 2019 Mar ^1st^, patients that diagnosed with bladder cancer were collected at Sun Yat-Sen Memorial Hospital.

## Abbreviations

CCK8: Cell Counting Kit-8
circRIP2: Hsa_circ_0005777
EMT: Epithelial-Mesenchymal Transition
FBS: Fatal Bovine Serum
FISH: Florescent in situ hybridization
RNA-seq: RNA-sequencing
U3: UM-UC-3
