# Correction to: circRIP2 accelerates bladder cancer progression via miR-1305/Tgf-β2/smad3 pathway

**DOI:** 10.1186/s12943-020-01284-5

**Published:** 2021-01-02

**Authors:** Yinjie Su, Weilian Feng, Juanyi Shi, Luping Chen, Jian Huang, Tianxin Lin

**Affiliations:** 1 The Department of Urology, Sun Yat-Sen Memorial Hospital, Sun Yat-Sen University, Guangzhou, China; 2 The Department of Endocrinology, Sun Yat-Sen Memorial Hospital, Sun Yat-Sen University, Guangzhou, China; 3 The Department of Pediatric Surgery, Sun Yat-Sen Memorial Hospital, Sun Yat-Sen University, Guangzhou, China

**Correction to: Mol Cancer 19, 23 (2020)**

**https://doi.org/10.1186/s12943-019-1129-5**

Following the publication of the original paper [1], the authors found two errors in the figures caused by incaution of compose type.

In Fig. 1a, the band of 5637 is similar to U3. After checking the original data, authors found that the pictures of 5637 and U3 cells were saved in the same file. When they disposed the pictures in Photoshop, the longer exposure picture of 5637 cells was mislabeled as U3 cells.

In Fig. 2i, it was confused the overlap of si1 and si2. After checking the original data, authors found the problem happened in the data acquisition. In the transwell assay, they took 10 pictures for each sample, but mislabeled the No.10 of si#1 as No.1 of si#2, resulting in other area of picture of si#1 cells was mistakenly reused as si#2 cells. In Fig. 2k, the mistake of repeated picture was caused by incaution of compose type. These errors have been corrected in the revised Fig. 2i and k as shown below.

The unintentional errors might cause misunderstand to the editor, reviewers and readers. The authors take full responsibility for this unintentional error. To better reduce the effect of these mistakes, several corrections were made as above. Since these errors do not originate from original experiments, and all the experiments have been repeated at least 3 times, there is no effect on the interpretation or conclusion of this work. Authors truly apologize for our overlook in this matter. They apologize to the editors, reviewers and readers for any confusion that was caused by this unintentional error.




