# Supplementary figures and images for: circRIP2 accelerates bladder cancer progression via miR-1305/Tgf-β2/smad3 pathway
Source: Mol Cancer. 2020 Feb 4;19:23. doi: 10.1186/s12943-019-1129-5 (PMC6998850; doi:10.1186/s12943-019-1129-5)

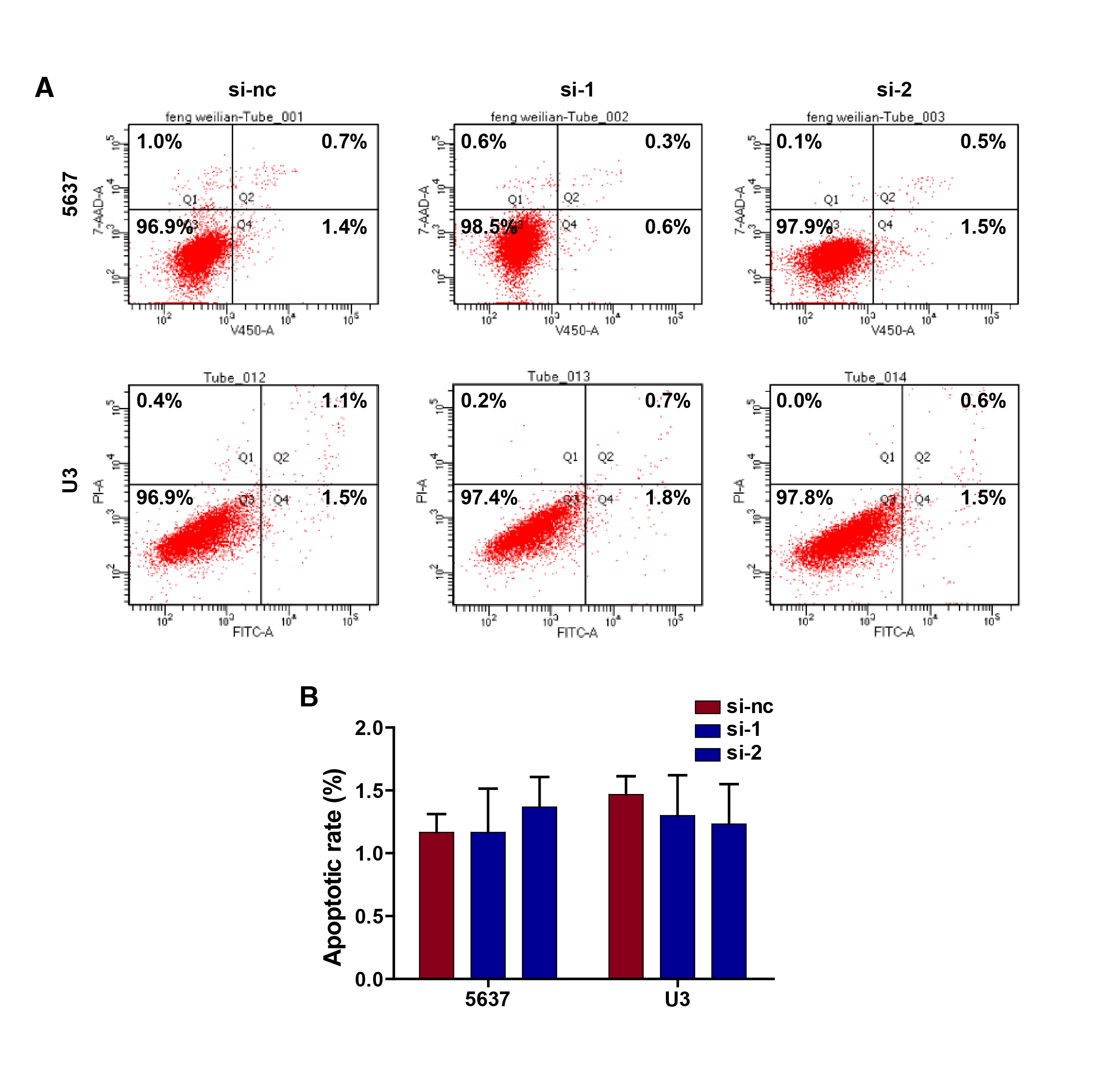

Supplement: Supplementary file 1 — Additional file 1: Figure S1. Silencing circRIP2 contributes less effect on bladder cancer apoptosis. A.B. Annexin V/Pi apoptotic assay was performed to detect effect of circRIP2 on the apoptosis of bladder cancer cells. [file 12943_2019_1129_MOESM1_ESM.tif]

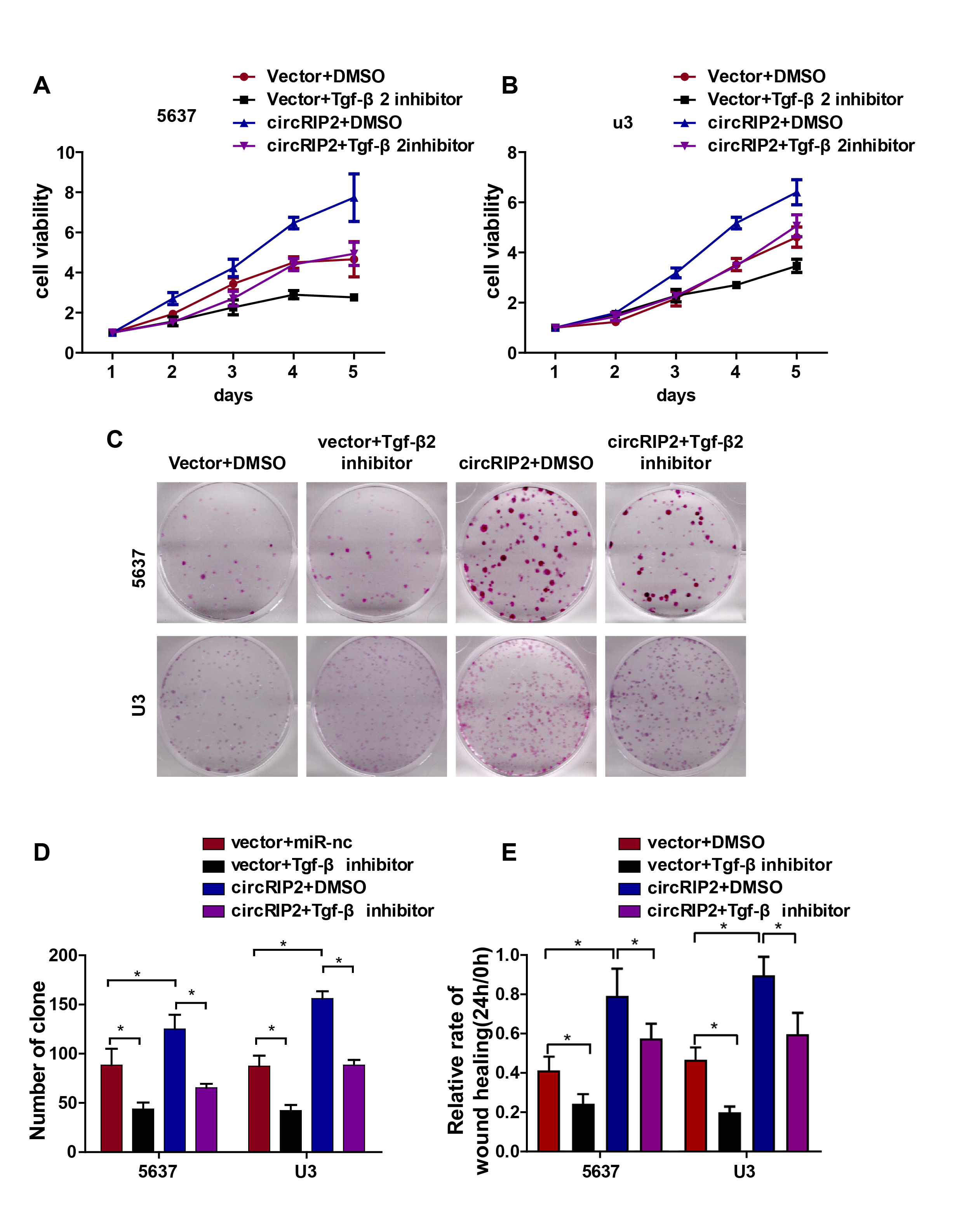

Supplement: Supplementary file 2 — Additional file 2: Figure S2. Tgf-β2 inhibitor revered cancer promotive role of circRIP2 in bladder cancer cells in vitro. A,B. CCK8 assay was performed to detect cell viability of bladder cancer cells; C,D. Cell potential to replicate and self-renew was reflected by clone formation; E,F. Rate of wound healing assay showed cell potential of migration; scale bar: 100μm. G,H,I,J,K. Trans-well migration and matrigel invasion assay showed cell potential of migration and invasion of bladder cancer cells; scale bar: 25μm. [file 12943_2019_1129_MOESM2_ESM.zip › 9-01.tif]

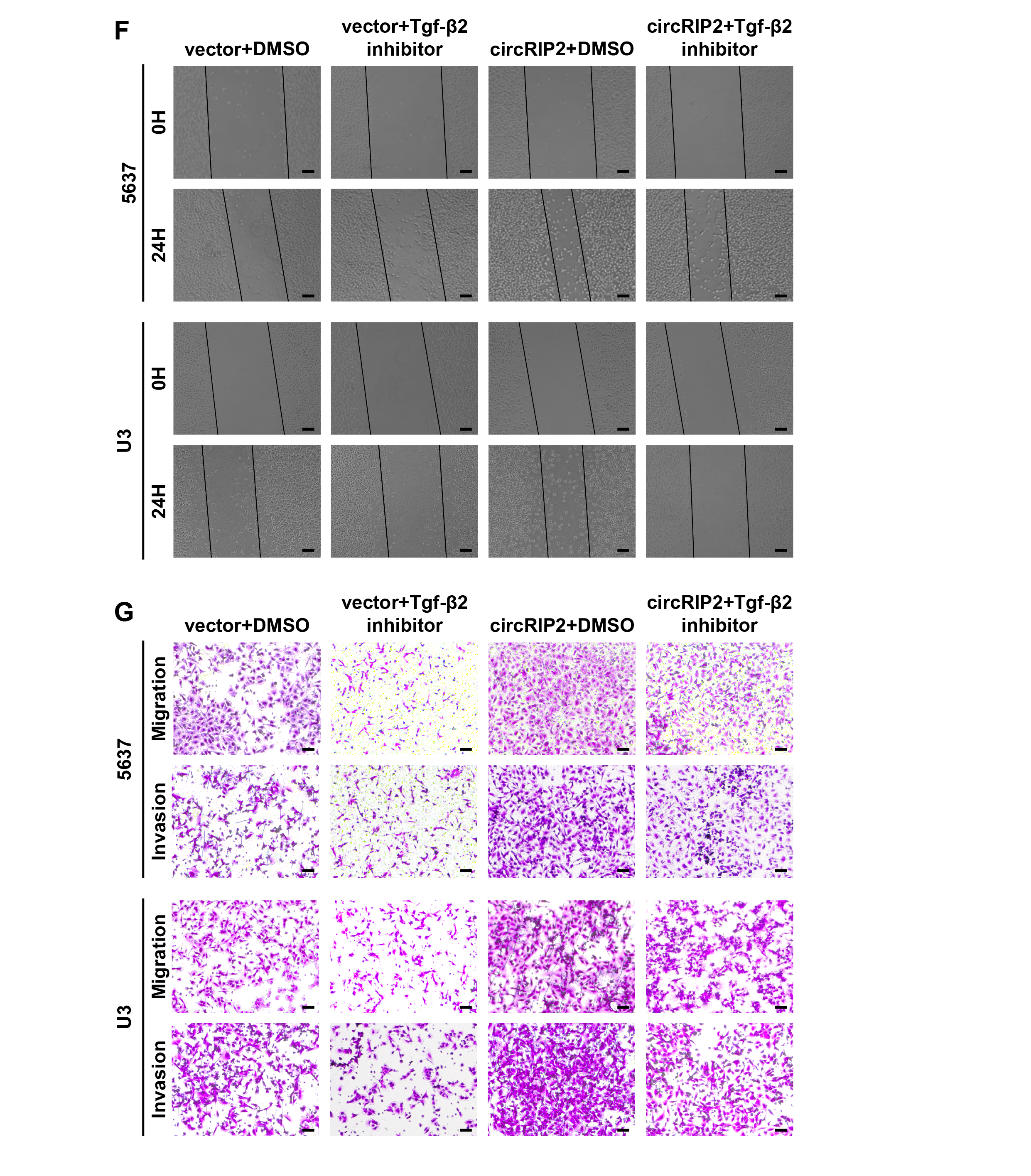

Supplement: Supplementary file 2 — Additional file 2: Figure S2. Tgf-β2 inhibitor revered cancer promotive role of circRIP2 in bladder cancer cells in vitro. A,B. CCK8 assay was performed to detect cell viability of bladder cancer cells; C,D. Cell potential to replicate and self-renew was reflected by clone formation; E,F. Rate of wound healing assay showed cell potential of migration; scale bar: 100μm. G,H,I,J,K. Trans-well migration and matrigel invasion assay showed cell potential of migration and invasion of bladder cancer cells; scale bar: 25μm. [file 12943_2019_1129_MOESM2_ESM.zip › 9-02.tif]

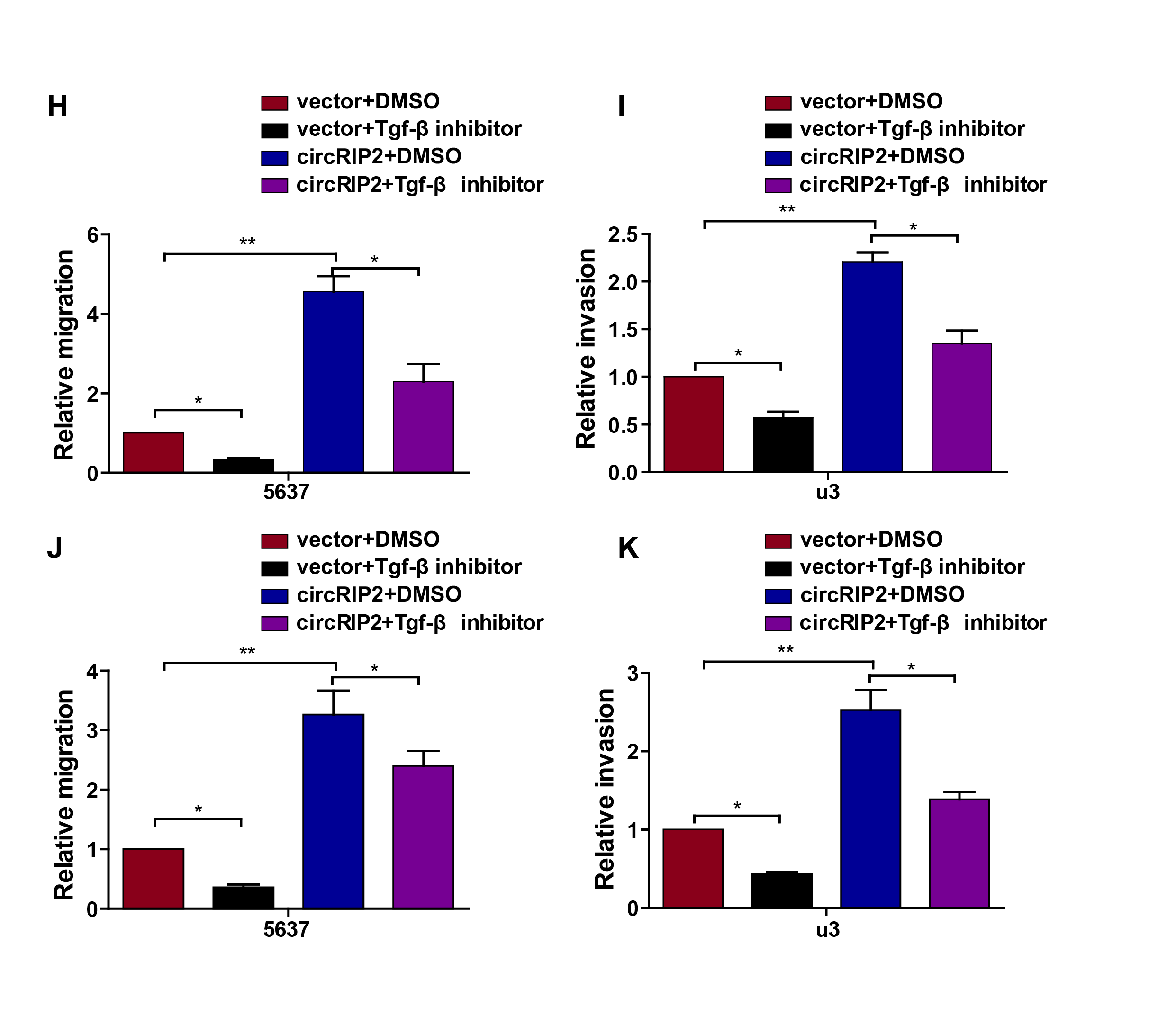

Supplement: Supplementary file 2 — Additional file 2: Figure S2. Tgf-β2 inhibitor revered cancer promotive role of circRIP2 in bladder cancer cells in vitro. A,B. CCK8 assay was performed to detect cell viability of bladder cancer cells; C,D. Cell potential to replicate and self-renew was reflected by clone formation; E,F. Rate of wound healing assay showed cell potential of migration; scale bar: 100μm. G,H,I,J,K. Trans-well migration and matrigel invasion assay showed cell potential of migration and invasion of bladder cancer cells; scale bar: 25μm. [file 12943_2019_1129_MOESM2_ESM.zip › 9-03.tif]

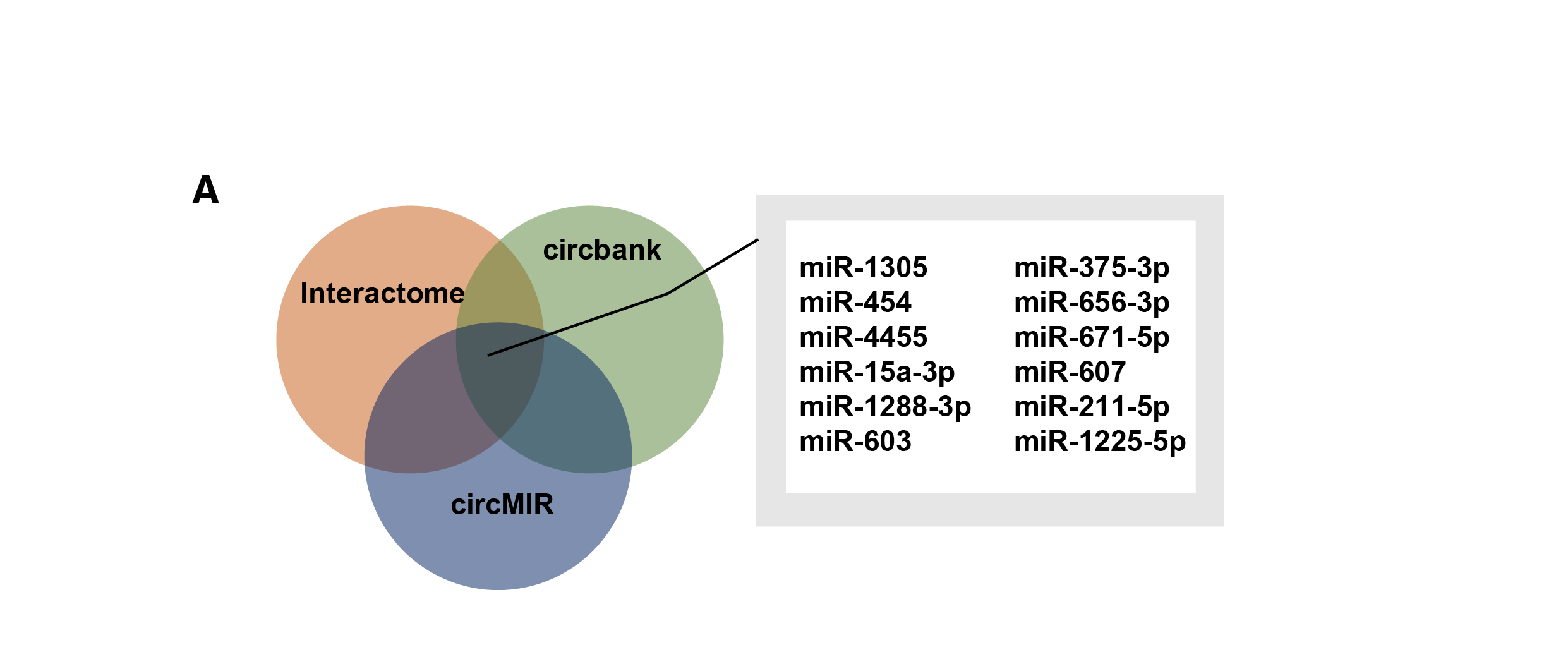

Supplement: Supplementary file 3 — Additional file 3: Figure S3. miRNAs that may bind with circRIP2 was predicted. 12 overlapped miRNAs were predicted from Interactome, circbank and circMIR together. [file 12943_2019_1129_MOESM3_ESM.tif]

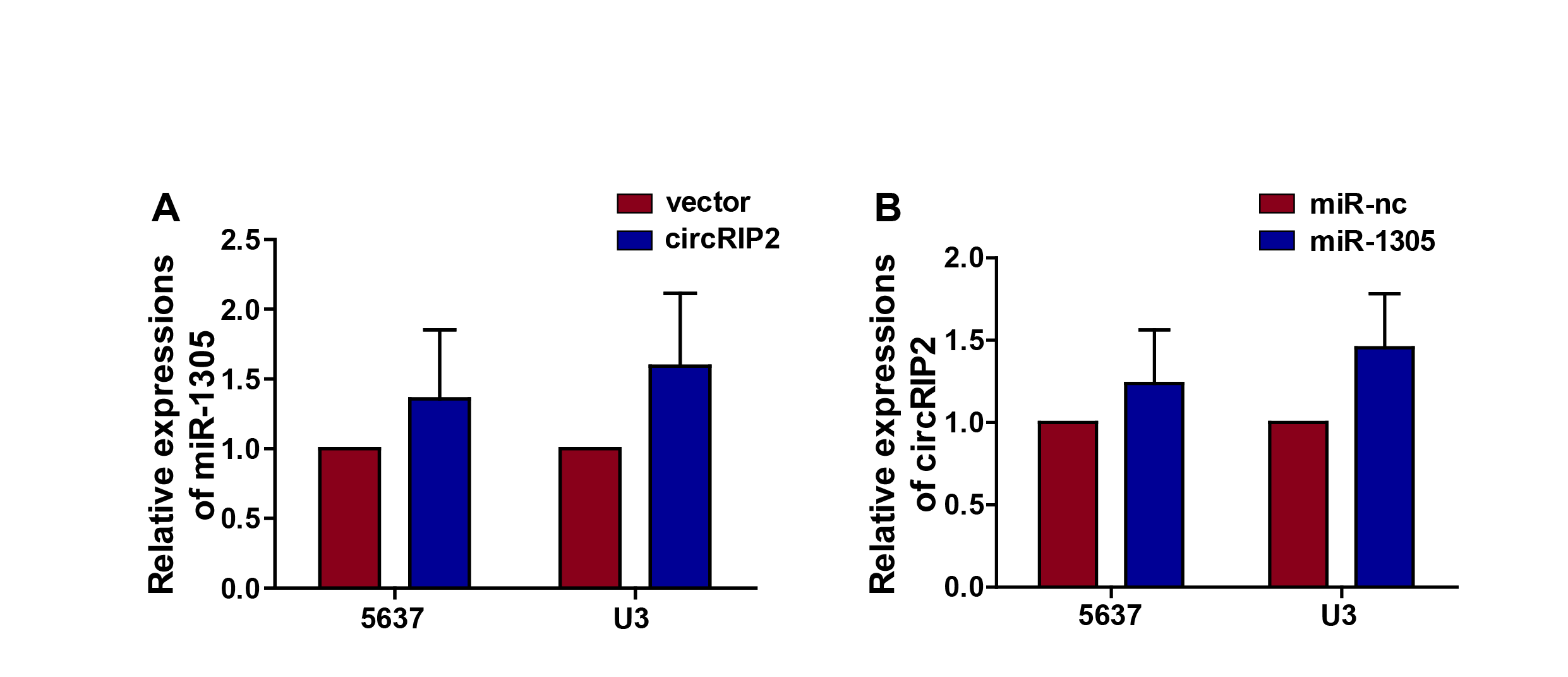

Supplement: Supplementary file 4 — Additional file 4: Figure S4. Expression of miR-1305 and circRIP2 was detected under each over-expression. A.B qPCR was used to detect expression of circRIP2 or miR-1305. [file 12943_2019_1129_MOESM4_ESM.tif]

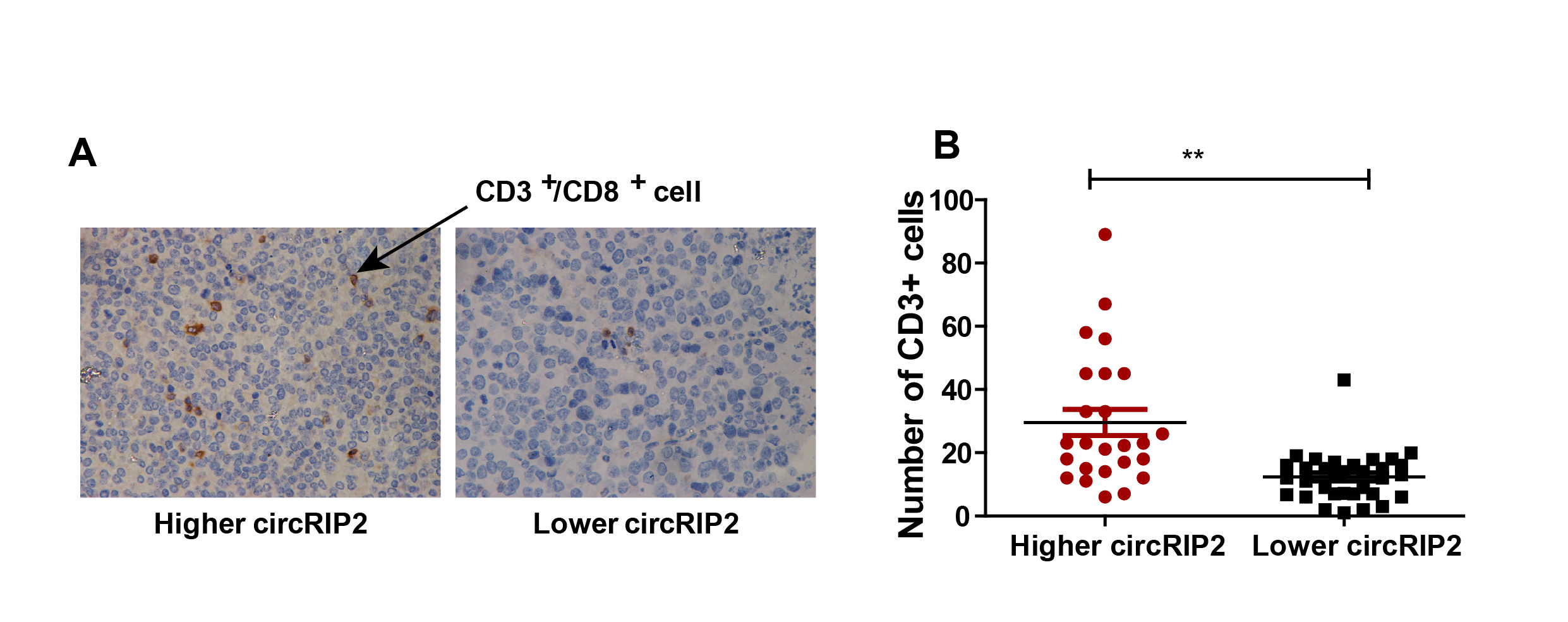

Supplement: Supplementary file 5 — Additional file 5: Figure S5. Higher circRIP2 patients display stronger immune infiltration. A,B Immune histochemistry detected infiltration of CD3 and CD8 cells among paraffin-embedded tissues. Cells in 10 randomly selected views were counted. Views were photographed under 200× microscopically. [file 12943_2019_1129_MOESM5_ESM.tif]
