# Supplementary material for: circRIP2 accelerates bladder cancer progression via miR-1305/Tgf-β2/smad3 pathway
Source: Mol Cancer. 2020 Feb 4;19:23. doi: 10.1186/s12943-019-1129-5 (PMC6998850; doi:10.1186/s12943-019-1129-5)
Supplement: Supplementary file 6 — Additional file 6: Table S1. List of primers for qPCR. Table S2. List of sequences for siRNAs. Table S3. List of probe sequences for RNA pulldown. [file 12943_2019_1129_MOESM6_ESM.docx]

Table EV1 List of primers for qPCR

| Gene | Sequence(5’-3’) |
| --- | --- |
| circRIP2 | F: CACCATCAAGTTCGTTTGCT |
|  | R: GGCTGGTAGTGGCAGTGATT |
| RIP2 | F: CCCATCTGGAATTGCCTCTA |
|  | R: CACCCTCTTCGTTGGGTAAA |
| GAPDH | F: TACTAGCGGTTTTACGGGCG |
|  | R: TCGAACAGGAGGAGCAGAGAGCGA |
| miR-1305 | F: CTCAACTGGTGTCGTGGAGTCGGCAATTCAGTTGAGCAGTAGAG |
|  | R: ACACTCCAGCTGGGGTGTTGAAACAATCT |
| U6 | F: CGCTTCGGCAGCACATATAC |
|  | R: TTCAGAATTTGCGTGTCAT |

Table EV2. List of sequences for siRNAs

| siRNA | Sequence(5' -3') |
| --- | --- |
| circRIP2 si-1 | sense: CUAAAGAAAAGAAGGUUGGTT |
|  | antisense: CCAACCUUCUUUUCUUUAGTT |
| circRIP2 si-2 | sense: GAAAAGAAGGUUGGUGAUUTT |
|  | antisense: AAUCACCAACCUUCUUUUCTT |

Table EV3 List of probe sequences for RNA pulldown

| Gene | Sequence(5’-3’) |
| --- | --- |
| circRIP2 (5’biotin) | GAUAUCCAAAUCACCAACCUUCUUUUCUUUAGAUGAC |
| Oligo (5’biotin) | GGUACUGGAUAUAGUACAGAUCCAGUCCUUCUGUGGA |
| miR-1305 (5’biotin) | UUUUCAACUCUAAUGGGAGAGAUCUCCCAUUAGAGUUGAGGGUU |
| miR-nc (5’biotin) | UUCUCCGAACGUGUCACGUTTACGUGACACGUUCGGAGAATT |
